# Interactions of SARS-CoV-2 with the Blood–Brain Barrier

**DOI:** 10.3390/ijms22052681

**Published:** 2021-03-06

**Authors:** Michelle A. Erickson, Elizabeth M. Rhea, Rachel C. Knopp, William A. Banks

**Affiliations:** 1 Geriatric Research Education and Clinical Center, VA Puget Sound Healthcare System, Seattle, WA 98108, USA; meredime@uw.edu (E.M.R.); rcknopp@uw.edu (R.C.K.); 2 Division of Gerontology and Geriatric Medicine, Department of Medicine, University of Washington School of Medicine, Seattle, WA 98104, USA

**Keywords:** blood–brain barrier, SARS-CoV-2, COVID-19, brain, inflammation, thrombosis, hypoxia, APOE

## Abstract

Emerging data indicate that neurological complications occur as a consequence of severe acute respiratory syndrome coronavirus 2 (SARS-CoV-2) infection. The blood–brain barrier (BBB) is a critical interface that regulates entry of circulating molecules into the CNS, and is regulated by signals that arise from the brain and blood compartments. In this review, we discuss mechanisms by which SARS-CoV-2 interactions with the BBB may contribute to neurological dysfunction associated with coronavirus disease of 2019 (COVID-19), which is caused by SARS-CoV-2. We consider aspects of peripheral disease, such as hypoxia and systemic inflammatory response syndrome/cytokine storm, as well as CNS infection and mechanisms of viral entry into the brain. We also discuss the contribution of risk factors for developing severe COVID-19 to BBB dysfunction that could increase viral entry or otherwise damage the brain.

## 1. Introduction

The world is entering a new phase of the severe acute respiratory syndrome coronavirus 2 (SARS-CoV-2) pandemic. The increasing availability of highly efficacious vaccines will eventually begin to counter the rising numbers of coronavirus disease of 2019 (COVID-19) cases, resulting in lives saved, disabilities prevented, and burdens on healthcare systems mitigated. As we look forward to the future, we also reflect on the current tragedy of over a million lives lost so far and the many more survivors of infection who face difficult recoveries. SARS-CoV-2 has proven to be an enigmatic virus, in that there is a wide array of clinical presentations following infection, ranging from no symptoms to severe, life-threatening disease and death. Further, although SARS-CoV-2 is predominantly a respiratory virus, it can cause dysfunction of organs outside the lungs including the kidneys, heart, liver, and brain [1,2,3]. Neurological complications of SARS-CoV-2 infection can be serious and debilitating and have contributed to a decreased quality of life in a substantial portion of the population in recovery from SARS-CoV-2 infection. Currently, there is evidence that SARS-CoV-2 can contribute to neurological dysfunction through mechanisms that include infection of CNS resident cells, which would involve viral entry into the brain, and systemic activation of the immune system, which does not necessarily involve viral entry into the brain, but rather pathophysiological interactions between the brain and immune system. The vascular blood–brain barrier (BBB) is an important brain interface that regulates the passage of substances between the blood and brain compartments and has additional mechanisms for regulating neuroimmune communication. The BBB is thus an important substrate for direct and indirect SARS-CoV-2 interactions with the brain. In this review, we evaluate the participation of the BBB in SARS-CoV-2 infections that could contribute to COVID-19-associated neurological dysfunctions.

### 1.1. Structure and Functions of the BBB

**Figure 1:**
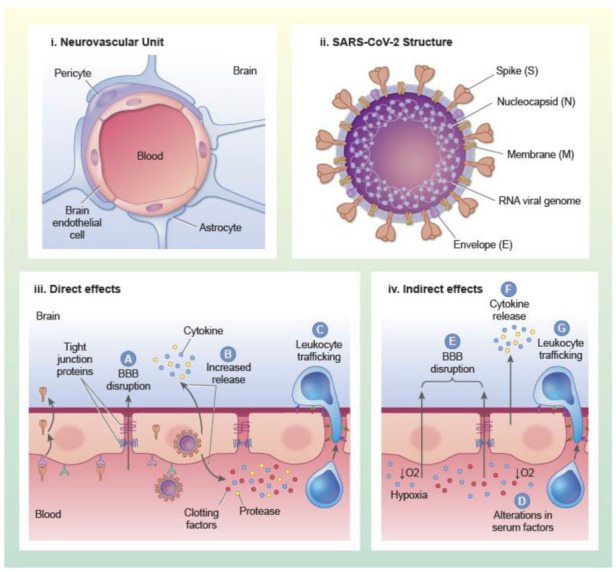
Impact of SARS-CoV-2 on the BBB structure and function. (**i**) Model of the neurovascular unit showing brain endothelial cells, pericytes, and astrocytes. (**ii**) Model of SARS-CoV-2 virion structure. (**iii**) Proposed direct effects of SARS-CoV-2 on the BBB. (**A**) BBB disruption that occurs due to SARS-CoV-2 protein interactions with the brain endothelial cell can cause non-specific leakage of serum factors into the brain. (**B**) SARS-CoV-2 protein interactions with brain endothelial cells may cause the release of cytokines, proteases, or clotting factors into the blood or brain compartments, as well as (**C**) increased expression of cell adhesion molecules which could contribute to leukocyte trafficking. (**iv**) Proposed indirect effects of SARS-CoV-2 on the BBB. (**D**) SARS-CoV-2 infection can increase circulating concentrations of pro-inflammatory cytokines and clotting factors, and decrease oxygen levels which may induce (**E**) BBB disruption via paracellular or transcellular routes, (**F**) increased production and release of cytokines and proteases by the brain endothelium, and (**G**) upregulation of brain endothelial cell adhesion molecules and leukocyte trafficking to the brain.

The primary unit of the vascular BBB is the brain endothelial cell (BEC), and BECs are associated with other cell types such as pericytes and astrocytes that support BBB induction and maintenance (Figure 1). BECs have a specialized phenotype that contributes to the regulation of substrate transfer into and out of the CNS. The barrier properties of BECs are conferred, in part, through expression of tight junction protein complexes that localize to endothelial cell–cell junctions and form a tight paracellular barrier that prevents the diffusion of substances between cells. Tight junction protein complexes at the BBB are comprised of occludens, claudins, and junctional adhesion molecules such as zonula occludens [4,5]. Claudins are a large protein family of transmembrane proteins with four membrane spanning domains. A recent comprehensive survey of claudin expression in laser capture dissected brain microvessels from mice and humans showed that claudins 1–6, 9, 11, 12, 14, 15, 17, 20, 22, 24, and 25 were detected at the mRNA level, and that claudins 5 and 25 were the most abundant at the protein level [6]. Claudin 5 predominantly regulates paracellular leakage at the BBB to molecules under about 800 Da [7]. Tight junctions limit not only the paracellular diffusion of substances between endothelial cell junctions, but also the lateral diffusion of membrane proteins. Therefore, tight junctions also confer polarity to BECs. Tight junction proteins interact with the cytoskeletal components, adherens junctions, and the extracellular matrix, and are regulated by a variety of physiological and pathophysiological stimuli [4]. BECs also suppress their own formation of fenestrae and vesicular structures such as macropinocytic vesicles. The lack of fenestrae and suppression of macropinocytosis suppresses transcellular leakage of circulating substances into the brain. The regulation of vesicular processes at the BBB is understudied, but recent works have revealed key regulators of vesicle formation that are uniquely active in BECs. The lipid transporter major facilitator superfamily domain containing 2A (Mfsd2a), for example, prevents the formation of caveolin-1 vesicles by regulating the lipid composition of brain endothelial cell membranes [8,9]. Other barrier properties of BECs are conferred through expression of efflux transporters such as P-glycoprotein and metabolic enzymes which can limit the CNS entry of xenobiotics and endogenous substrates [10,11].

Another critical function of the BBB is to provide the brain with nutritive and trophic support from the circulation. However, many of the circulating substrates that are required for proper CNS function cannot freely diffuse across BEC membranes, and so require transporters at the BBB to permit their passage from brain-to-blood. Similarly, peptides and proteins, including some cytokines and chemokines, utilize specialized transport systems to cross the BBB and to a more limited degree cross by way of transmembrane diffusion. Transport systems at the BBB include energy-independent systems such as solute carriers, which facilitate transport of molecules such as glucose and amino acids down a concentration gradient. Other transport processes are energy-dependent, and involve vesicular mechanisms such as receptor-mediated transcytosis and adsorptive endocytosis [12]. BBB transporters are important for relaying signals to the brain that facilitate monitoring of physiological states such as inflammatory status [13]. In addition to regulating transport, the BBB also functions as an important signaling and secretory interface, which facilitates bidirectional communication between the brain and blood compartments [13]. In a later section, we will discuss these aspects of BBB functions in context of the passage of SARS-CoV-2 into the brain, as well as the neuroinflammatory response to SARS-CoV-2 infections.

The phenotypic specialization of BECs depends on signals from their local environment, which includes signals from closely associated supportive cells such as astrocytes, pericytes, neurons, microglia, oligodendrocytes, and others [13]. Collectively, BECs, their associated cells, and extracellular matrix components that regulate BEC functions are referred to as the neurovascular unit (NVU) [14,15]. Of the cell types that are considered part of the NVU, pericytes are the most closely associated with BECs, being located primarily around capillaries and post-capillary venules. Pericytes and BECs share a basement membrane and cytoplasm through gap junctions. The predominant functions that have been ascribed to pericytes at the BBB include induction and maintenance of the barrier phenotype as well as regulation of microvascular tone [9,16,17]. The endfeet of astrocytes are also closely associated with BECs, and ensheath the brain microvasculature. Astrocytes are important for inducing and maintaining the BBB phenotype and contribute to the regulation of capillary tone through cross-talk with pericytes [18,19]. Astrocytes, pericytes and other components of the NVU contribute to BBB functions under physiological conditions and in inflammatory states, which has recently been reviewed [13]. We will consider in later sections how SARS-CoV-2 infection could influence cross-talk among cells of the NVU to modulate BBB functions.

### 1.2. Neurological Complications of SARS-CoV-2 Infection and BBB Involvement

A diverse range of neurological complications have been associated with SARS-CoV-2 infection, and our understanding of causal and mechanistic relations is evolving. Headache, anosmia and dysgeusia are among the most prevalent neurological symptoms of SARS-CoV-2 infection [20,21,22], and these symptoms typically resolve over time in most mild cases [23]. However, more serious neurological complications such as impaired consciousness, cerebrovascular events, encephalopathy/encephalitis, acute disseminated encephalomyelitis, Guillain-Barré syndrome, seizures, delirium, dementia-like syndrome, and psychiatric disorders including psychosis, catatonia and mania have been reported [2,3,24,25]. There is also a phenomenon of chronic sequelae of COVID-19 that affects a proportion of infected individuals, currently termed “Long COVID” or “Long-haul COVID” that includes neurological symptoms [26,27]. Recent work that evaluated anxiety and depression and fatigue/muscle weakness 6 months post-infection found that disease severity increased the risk for the persistence of these outcomes. Female sex was also associated with greater risk for prolonged symptoms of anxiety and depression [28]. Notably, most of the current information available on the onset and prevalence of neurological symptoms of COVID-19 is limited to hospitalized patients and comprehensive evaluation of non-hospitalized patients currently is limited or lacking.

The neurological manifestations of SARS-CoV-2 infection could be attributed to direct effects of the virus on the nervous system, para- or post-infectious disease mediated by the immune system, coagulopathies, and/or complications of critical illness [3,29]. In each of these scenarios, unique aspects of BBB involvement can be considered and we will explore these factors in later sections of the review.

## 2. Mechanisms of SARS-CoV-2 Infection and Tissue Tropism

### 2.1. Virus Structure

Coronaviruses are large, enveloped, positive-stranded RNA viruses that can infect a wide array of mammalian and avian species. There are currently seven coronaviruses that can infect humans (HCoV), and the betacoronaviruses MERS-CoV, SARS-CoV-1, and SARS-CoV-2 can cause severe disease in humans. Other coronaviruses HCoV-229E, OC43, NL63, and HKU1 generally cause mild upper-respiratory tract illness including the common cold [30]. SARS-CoV-2, like other coronaviruses, possesses four main structural proteins: nucleocapsid (N) protein, membrane (M) glycoprotein, small envelope (E) glycoprotein, and the spike (S) glycoprotein, and the arrangement of these in the virion are shown in Figure 1. These proteins are responsible for virion assembly, virus–host cell receptor binding, and release of viral particles from the host cell [31]. The heavily phosphorylated N protein is found encapsulating the positive-sense, single-stranded RNA genome and has roles related to viral replication [32]. The M glycoprotein is the most abundant protein and spans the membrane bilayer, leaving a short NH2 domain outside the virus and a long COOH terminus in the viral particle [33]. M protein can bind to the other SARS-CoV-2 structural proteins, and contributes to stability of the virion. For example, binding with M helps to stabilize N proteins and promotes completion of viral assembly by stabilizing the N protein-RNA complex inside the virion [34]. Found in the viral membrane, the E protein is the smallest and has roles in viral maturation and production [35,36]. E glycoprotein binding with M and N glycoproteins helps to facilitate the virus-like particle formation.

Perhaps the most well-studied and characterized SARS-CoV-2 protein is the S glycoprotein, which initiates viral infection by interacting with host receptors and proteases [37]. S is a transmembrane protein which forms the spike-like projections that protrude from the virus. S consists of a short cytoplasmic region, a transmembrane domain, and two extracellular subunits, S1 and S2. These subunits are produced through proteolytic cleavage by the host cell enzyme furin during intracellular processing. The S1 subunit contains the receptor-binding domain (RBD) and is responsible for the determination of the host virus range and cellular tropism, whereas the S2 subunit makes up the stalk of the spike molecule, and functions to mediate virus fusion in host cells. The RBD of the S protein has been an important target for SARS-CoV-2 therapies and vaccines [38].

### 2.2. Host Receptors and Other Factors That Mediate SARS-CoV-2 Entry into Cells

The initial steps of SARS-CoV-2 infection involve receptor binding and fusion of the viral lipid envelope with cellular membranes. Recent reports have shown that the host receptor that is primarily responsible for binding SARS-CoV-2 is angiotensin-converting enzyme 2 (ACE2), which is also the receptor for SARS-CoV-1 [39,40,41]. To engage the ACE2 receptor, the RBDs of S1 undergo hinge-like movements to form a concave surface which allows for binding with the N-terminal alpha helix of ACE2. The domains are held together primarily through polar interactions, with a robust network of hydrogen bonds and salt-bridges [42]. Fusion of the viral and host membranes is facilitated by cleavage of the S protein into its subunits, S1 and S2. Cleavage at two sites, S1/S2 and S2′, on the S protein are required for fusion. These cleavages are mediated by furin at the S1/S2 site, and type II transmembrane serine protease (TMPRSS2) at the S2′ site [43,44,45]. Other host proteins may further influence the infectivity of the virus. For example, Neuropilin-1 (NRP-1) binds S1 following furin cleavage, which promotes viral entry and infection [46]. Cathepsin B/L was shown to facilitate S cleavage and promote infection [41]. Other host proteins have been reported to facilitate SARS-CoV-2 entry, including basigin, although its role in binding S is controversial [47,48]. SARS-CoV-2 S protein can also bind sialic acids which are often important in the glycation of the cell glycoproteins used by viruses to bind to cells; however it is not known to what extent this interaction contributes to infection [49]. The cell-type specific expression of proteins and other molecules that are involved in SARS-CoV-2 binding and fusion are likely to contribute to tissue tropism [50], which is discussed in the next section.

### 2.3. Tropism of SARS-CoV-2: Evidence for Direct Infection of the CNS and Implications for Disease

Data in humans and animal models suggest that SARS-CoV-2 primarily infects cells in the respiratory tract but can also spread to other organs. RT-qPCR based methods that detect viral mRNA are the most sensitive methods used to detect the presence of SARS-CoV-2 in tissues, and viral mRNA has been detected outside of the respiratory tract including in the conjunctiva, heart, immune cells, blood and plasma, stool and rectal swabs, urine, kidney tissue, semen, breast milk, cerebrospinal fluid, and in brain biopsies [51]. Table 1 summarizes the studies that have attempted to detect SARS-CoV-2 in human brain tissue and CSF, and associated neuropathological findings. Importantly, the detection of viral mRNA does not necessarily indicate the presence of infectious virus, and detection of infectious virus outside the respiratory tract has been more limited to stool and urine in humans [51]. Since ACE2 is thought to be the predominant receptor that mediates binding of SARS-CoV-2 to cells, it has been predicted that ACE2 expression is a determinant of SARS-CoV-2 cellular tropism. This prediction is supported, in part, by findings in ACE2 transgenic mice. Wild-type mouse strains are poor hosts for SARS-CoV-2, however, mice that overexpress a human form of ACE2 develop productive infections and clinical disease [52]. ACE2 is highly expressed on human lung alveolar epithelial cells and intestinal enterocytes, nasal and oral mucosa, and on arterial and venous endothelial cells and arterial smooth muscle cells in organs such as the lungs, intestines, skin, spleen, and brain [53]. Further, SARS-CoV-2 viral-like particles have been detected inside endothelial cells by electron micrographs of autopsied patient tissues, and in at least one instance cell membrane blebs containing viral-like particles were observed in brain endothelial cells [54,55]. However, it is not clear whether the presence of virus inside the endothelial cell reflects viruses that are replicating or viruses that have only entered tissues. In vitro, it has been shown that SARS-CoV-2 can infect human blood vessel organoids in an ACE2-dependent manner [56]. A survey of primary human endothelial cells from tissues including brain found that SARS-CoV-2 did not infect endothelial cells, which was attributed to the absence of ACE2 expression in vitro. Endothelial cells that overexpressed ACE2 via lentiviral vectors could support a productive infection [57], further indicating that SARS-CoV-2 infection of endothelial cells is ACE2-dependent.

Recent investigations support that SARS-CoV-2 can enter and infect the brain, and our understanding of how this occurs, what proportion of COVID-19 cases are affected, and the clinical relevance of CNS viral entry is evolving. COVID-19 patients who present with neurological symptoms often do not have detectable virus in their CSF, despite having other CSF and MRI brain abnormalities [58,59,65]. However, SARS-CoV-2 has been detected in brain tissue [51,60,61,62], and in one instance was detected in brain tissue and not CSF [55], suggesting that an absence of detection in CSF does not necessarily rule out brain infection. Mouse models that support SARS-CoV-2 infection and clinical disease through transgene-driven expression of human ACE2 can support productive brain infections [61,66], and ACE2 expression in the brain may contribute to severe clinical symptoms [61]. These results are summarized in Table 2. In a mouse with detectable SARS-CoV-2 neuroinvasion, meningeal inflammation and parenchymal infiltration of leukocytes was apparent, as was microgliosis. The same study reported that mouse brains without detectable SARS-CoV-2 neuroinvasion appeared to be normal [66]. Studies evaluating the cellular localization of SARS-CoV-2 signal in the brain have reported the presence of virus in endothelial cells and neurons [53,55,56,61,67], whereas infection of glial cells such as astrocytes and microglia has not been observed in vivo. Evidence supports that SARS-CoV-2 infection can cause damage and apoptosis of endothelial cells and neurons [61,68]. In ACE2-overexpressing mice, SARS-CoV-2 infection was limited to neurons and not evident in brain endothelial cells, although neurovascular abnormalities appeared in proximity to infected brain regions [61]. SARS-CoV-1 is also predominantly neurotropic in ACE2 overexpressing mice, and induces neuronal apoptosis without evidence of much gliosis, although elevations in pro-inflammatory cytokines in the brain are detected [69]. Importantly, ACE2 gene expression in mice driven by an ectopic promoter such as K18 should be carefully interpreted. In another mouse model where human ACE2 was knocked in under control of the mouse ACE2 promoter, virus was detected in brains by PCR but it was not determined whether this was replicative virus or whether the mice had brain pathology [70]. In summary, there is evidence of brain SARS-CoV-2 entry and infection, which could contribute to neuronal and endothelial damage and loss.

## 3. Mechanisms of BBB Dysfunction in SARS-CoV-2 Infection

### 3.1. Direct Interactions of SARS-CoV-2 with Brain Endothelial Cells and Other Constituents of the NVU

As described in the previous section, evidence suggests that SARS-CoV-2 might infect brain endothelial cells, and so dysfunction to the BBB could occur in response to infection or other direct interactions of SARS-CoV-2 or its components with the BBB. Some of these aspects of SARS-CoV-2 mediated brain endothelial dysfunction are illustrated in Figure 1. Presently, neurons are the only other NVU component that have been shown to be infected with SARS-CoV-2 and neuronal infection has been associated with neurodegeneration and neurovascular remodeling [61]. It has not yet been determined whether SARS-CoV-2 infects, binds, or directly alters function of non-endothelial NVU components. A recent study using primary human in vitro BBB models has shown that components of the SARS-CoV-2 spike protein, including S1, S2, and the receptor binding domain can all cause BBB leakage in the absence of toxicity [71]. Induction of BBB leakage occurred in response to glycosylated and non-glycosylated forms of S1 and S2 [71]. Infection of primary human endothelial cells that overexpressed ACE2 with SARS-CoV-2 induced the overexpression of clotting factors, adhesion molecules, and pro-inflammatory cytokines as well as formation of multinucleate syncytia and endothelial cell lysis [57]. Together, these data suggest that SARS-CoV-2 infection and contact with viral proteins could contribute to brain endothelial dysfunction and damage.

### 3.2. Indirect Effects of SARS-CoV-2 Infection on the BBB

#### 3.2.1. Inflammation

SARS-CoV-2 infection causes a systemic inflammatory response that results in the elevation of pro-inflammatory cytokines, chemokines, acute phase proteins, complement, and modification of leukocyte profiles in the brain and blood [72,73,74]. The BBB has the ability to respond to signals from the immune system that arise from the brain and blood compartments and can also regulate the transit of immune signals across compartments. Therefore, aspects of BBB dysfunction that may occur in response to SARS-CoV-2 infection could depend on interactions with the immune system in addition to or independently of interactions with the virus and its components. Figure 1 depicts mechanisms by which inflammatory factors, as well as related processes of hypercoagulation and hypoxia (described below) can contribute to BEC dysfunction. We have previously described immune functions and interactions of the BBB as five axes. These include (1) modulation of BBB leakage and (2) regulation of BBB transport functions and secretions by the immune system, (3) BBB transport and uptake of immunoactive substances, (4) BBB secretion of immunoactive substances, and (5) immune cell trafficking [13,75]. In this section, we will discuss what is known and what remains to be learned about the involvement of neuroimmune axes of the BBB in SARS-CoV-2 infections. We will also discuss how thrombotic-related complications may contribute to SARS-CoV-2- mediated BBB damage.

Systemic inflammation is a feature of SARS-CoV-2 infection. Elevations in plasma cytokines including IL-1β, IL-1RA, IL-7, IL-8, IL-9, IL-10, bFGF, GCSF, GM-CSF, IFN-γ, IP-10, CCL2, CCL3, CCL4, PDGF, TNF-α, and VEGF have been reported in COVID-19 cases requiring hospitalization for pneumonia, and IL-2, IL-7, IL-10, G-CSF, IP-10, CCL2, CCL3, and TNF-α were shown to be higher in patients admitted to the ICU [72]. IL-6 and D-dimer were also shown to be greater in COVID-19 non-survivors vs. survivors [73], and higher levels of the acute phase proteins CRP and SAA in plasma have been associated with more severe disease and poorer outcomes in patients with COVID-19 [76,77]. Elevated cytokines have also been detected in the CSF of COVID-19 patients with neurological presentation [78,79,80], indicative of neuroinflammation. Cytokines, chemokines, and acute phase proteins are involved in the 5 neuroimmune axes [13,75,81]. For example, cytokines like IL-1β, IL-6, TNF-α, IFN-γ, chemokines like CCL2, and the acute phase protein CRP can cause BBB disruption and can modulate the functions of BBB transporters like P-glycoprotein and can influence adsorptive transcytosis which is one mechanism by which HIV-1 can cross the BBB [13,75,82,83]. Cytokines and chemokines including TNF-α, IL-1α, IL-1β, IL-1RA, IL-6, CCL2, and CCL11 are also substrates for transport across the intact BBB, and G-CSF, IL-6, IL-13, TNF-α, CXCL1, CCL2, CCL3, CCL4, CCL5, and CCL11 can be secreted by BECs and other cells of the NVU [13,75]. Leukocyte trafficking to the brain and CSF has been observed in SARS-Cov-2 infections and may be higher in fatal cases [84]. Vascular BBB involvement has been implicated, based on observations of infiltrating perivascular macrophages and CD3+ and CD8+ T-cells in the perivascular spaces adjacent to endothelial cells [63]. Markers of reactive gliosis have also been observed in proximity to brain endothelial cells in post-mortem COVID-19 brain tissue, suggesting that neuroinflammation may contribute to brain microvascular injury [63]. The CNS perivascular infiltration of immune cells and reactive gliosis that has been observed in some cases of SARS-CoV-2 infection also suggests changes in other non-endothelial components of the NVU. Immune cell trafficking across the BBB involves leukocyte interactions with and modifications of the basement membrane to permit entry into the brain parenchyma [85,86], although modifications of NVU basement membranes in vivo during diapedesis are generally not well-studied [86] and have not been evaluated in context of SARS-CoV-2 infection. SARS-CoV-2 can cause pericyte loss in the lungs in patients who develop severe COVID-19, which may precede vasculopathy [87]. Pericyte loss in the CNS has not yet been evaluated in SARS-CoV-2 infections.

Notably, the study of neurological changes as they relate to inflammatory changes in plasma, CSF, and brain tissues in SARS-CoV-2 cases has mostly been limited to more serious cases requiring hospital admission. However, the BBB may also be involved in symptom presentation in mild cases of SARS-CoV-2 infection. A common symptom of SARS-CoV-2 infection is fever, which occurs in a large percentage of all SARS-CoV-2 infections [88]. Induction of fever depends on the synthesis of prostaglandin E2, which is mostly derived from BECs following an inflammatory insult [89]. Sickness behaviors, which are adaptive neurobehavioral responses to infection, are also cytokine-dependent and involve neuroimmune communication pathways that may or may not involve the BBB. For example, IL-1 induces endothelial PGE2, which then through the HPA axis induces symptoms of discomfort and malaise [90]. Blood-borne IL-1α impairs memory processing by crossing the BBB preferentially at the posterior division of the septum [91]. Pathways that circumvent the BBB involve cytokine interactions with vagal nerves in the periphery, which then communicate signals to the brain. Cytokines in blood can also signal to the brain through circumventricular organs, which are areas of the brain with leaky vasculature and connect to the rest of the brain by afferent and efferent projections [92]. Inflammation-related depressive-like behaviors, which can persist after sickness behaviors resolve [93], depend in part on overproduction and transport of kynurenine across the BBB [94].

#### 3.2.2. Clotting and Thrombosis

COVID-19 is also associated with high rates of thrombosis and thrombotic-related complications, including strokes, which can occur even in healthy young people without prior comorbidities [95]. Post-mortem analysis of brains from COVID-19 subjects showed a very high prevalence of acute hypoxic/ischemic damage [62]. High rates of coagulation abnormalities have been reported in COVID-19 patients [96], including cerebral microemboli [97], and the summation of clinical results support that SARS-CoV-2 infection increases the risk of immune-activated, complement-mediated thrombotic microangiopathy (TMA) [98]. TMA results from endothelial cell damage to small blood vessels, and leads to hemolytic anemia, thrombocytopenia, and can cause organ damage [99]. Neurologic imaging of SARS-CoV-2 patients with abnormal mental status and evidence of upregulated coagulation factors showed abnormalities that were consistent with TMA [96]. The complement pathway has been implicated in TMA that arises from SARS-CoV-2 infection, with unregulated formation of the C5b9 membrane attack complex identified as a potential driver [100]. Although C5b9 has not yet been evaluated in brains of subjects with SARS-CoV-2, it has been associated with brain injuries such as traumatic brain injury and subarachnoid hemorrhage [101,102]. Increases in other factors that are involved in clot formation and degradation such as thrombin, [103,104] fibrinogen, [105,106] and the plasmin system, [107,108] can cause BBB disruption. Elevated prothrombin time and levels of fibrin degradation products have been reported in blood samples taken from SARS-CoV-2 patients, with non-survivors showing amplified levels compared to survivors [109,110].

#### 3.2.3. Hypoxia

Moderate to severe levels of hypoxia can occur as part of the clinical presentation of COVID-19. Moreover, asymptomatic patients without respiratory distress can present with significantly reduced oxygen levels [111]; this “silent hypoxemia” was identified early during the initial Wuhan outbreak [112], and is correlated with poor outcomes [113]. Thus, many clinicians and researchers suggest hypoxia is a marker of COVID-19 severity [114].

Hypoxia has profound effects on the BBB. Numerous in vitro and in vivo studies show that oxygen deprivation induces BBB disruption, which may be a trigger for subsequent CNS disease [115]. Hypoxia induces paracellular permeability, dysregulation of tight junction protein expression levels, and can induce basement membrane breakdown [116,117,118,119]. Furthermore, hypoxia can increase the non-specific vesicular transport in brain endothelial cells, as shown by increased blood-borne proteins in the brain [120,121,122]. In neonatal stroke models, it has been shown that BBB and white matter damage depends on interactions of IL-1 with the BBB and can be inhibited by antibodies that block IL-1 brain entry [123,124,125].

During hypoxic conditions, the transcription factor hypoxia-inducible factor-1 (HIF-1) is activated [126]. This leads to an increase in HIF-1α protein levels which promotes cell survival in a variety of different mechanisms including upregulation of HIF-1α dependent genes which promote glucose transport and oxygen delivery. Furthermore, HIF-1α can increase angiogenesis by amplifying levels of vascular endothelial growth factor (VEGF, the major pro-angiogenic factor) and inducible nitric oxide (NO) synthase, which are responsible for stimulating the survival, proliferation, and vascular permeability in endothelial cells. These cellular response mechanisms aim to adapt tissues to the hypoxic environment. For example, hypoxia and low glucose levels can facilitate upregulation of P-glycoprotein levels in brain endothelial cells, possibly due to upregulation of HIF-1 [127,128]. However, the accompanying pathophysiological mechanisms increase BBB permeability and cerebrovascular oxidative stress that, under chronic conditions, can irreversibly damage the neurovascular unit [129]. It is important to note that other underlying mechanisms of SARS-CoV-2 infection, including hypoxia-induced increase in HIF-1α, can exacerbate the massive cytokine release [130].

## 4. Mechanisms of SARS-CoV-2 Transit across the Vascular BBB: Lessons from Neurotropic Viruses

As discussed above, SARS-CoV-2 and its interactions with the CNS are of critical importance to the immediate outcome of COVID-19 cases and also likely its long-term sequelae. However, little is known at this writing regarding the mechanisms by which SARS-CoV-2 accesses the brain and so this section of the review will rely heavily upon extrapolating the general principles of viral uptake by the CNS.

Most viruses are apparently unable to cross the mammalian brain barriers or to gain access to the nervous system. There are, however, significant exceptions where viruses both gain entry to the nervous system and that entry is the basis for or a contributor to their effects on nervous system function. In some cases, the CNS effects of a virus are minor, in other cases the CNS effects are part of a larger syndrome (e.g., HIV-1), and in still other cases the CNS effects can produce a syndrome so significant as to define the virus’ worst outcomes (rabies, measles). Where SARS-CoV-2 lies on this spectrum is yet to be determined.

Although there is a highly informative “classical” literature regarding the mechanisms by which viruses can enter the brain, the total literature on this topic is surprisingly small. Although key viruses have been studied in one regard or the other, most viruses, including those that produce significant CNS effects, remain unstudied. For example, the search terms “virus” plus “blood-brain barrier” finds less than 3000 total publications, of which 199 publications were in 2020. A total 81 publications were on “coronavirus” of which 53 of those were published in 2020. This state of the literature means that whereas specific statements can be made about the actions of a virus, its antithesis often cannot be. For example, whereas it may be known that a specific virus can cross the vascular BBB, it may be unknown whether it crosses the choroid plexus. Below, we list some of the general tenets as currently understood of how viruses enter the brain.

### 4.1. Viral Entry by Retrograde Nerve Transmission

Some viruses enter the brain by infecting peripheral nerves and then spreading to the brain by a process termed retrograde nerve transmission. Rabies is perhaps the best known virus to enter brain by retrograde nerve transmission, and does so by infecting motor neurons at neuromuscular junctions [131]. Alpha herpes viruses also enter the nervous system through retrograde nerve routes, infecting autonomic and sensory neurons [132]. Evidence suggests that a gastrointestinal reovirus can enter the brain via the vagus nerve, a cranial nerve and so part of the CNS [133]. Viruses that enter brain directly from the nasal passages use the olfactory nerve which extends beyond the cribriform plate into the nasal passages and the trigeminal nerve which projects to the walls of the nasal passages. Chou and Dix state that these pathways are “considered of only secondary importance” and “is limited primarily to infections acquired under unusual circumstances”. The coronavirus mouse hepatitis virus can enter brain by way of the olfactory and trigeminal nerves [134].

### 4.2. Viral Entry across Brain Barriers

Prior to the work of Bodian [135] on poliovirus, impermeability of the brain barriers was thought to prevent hematogenous spread of virus to the CNS. The hematogenous route is now considered the main pathway for viral entry into the CNS. The best studied of the brain barriers are the vascular BBB and the choroid plexus, although some work exists for viral entry at the meningeal barriers. Some viruses are endotropic, such as some togaviruses, and so are able to first infect the endothelial cells comprising the BBB with subsequent dissemination into brain. Likewise, viruses can infect the choroid plexus [136]. Some coronaviruses or their viral products have been found in brain and have presumptive evidence of entry at the BBB [137,138] and meninges [139]. Some viruses cross the BBB as free viruses and others cross by way of infected immune cells. These two pathways are not mutually exclusive, as exemplified by HIV-1 [140]. Some coronaviruses, e.g., MERS-CoV-2 and SARS-CoV-1, can infect immune cells [22,141], which has led to speculation that SARS-CoV-2 may enter the brain via infected immune cells [22]. When infected immune cells cross the BBB, they do so by a highly regulated mechanism that involves intercellular crosstalk between the immune cell and the endothelial cell, resulting in diapedesis, the physiological mechanism used by immune cells to enter the brain [142,143]. Immune cells normally traffic into brain at very low levels [144]. In the case of HIV-1, infected cells cross no better than uninfected cells, but both infected and uninfected cells both cross more rapidly because of the inflammatory status of AIDS [145].

### 4.3. BBB Disruption Plays a Selective Role in Viral Entry

BBB disruption is often cited as a mechanism by which viruses and infected immune cells can enter the CNS. The term BBB disruption is often used loosely to refer to many types of BBB dysfunction. Its more specific meaning relates to the loss of the ability of the vascular BBB to prevent the unregulated leakage of plasma and its contents into brain. The degree of BBB disruption varies greatly among the viruses, with HIV-1 being associated with relatively mild disruption and herpes simplex resulting in disruption that can raise CSF albumin levels to 150 mg/dl [146], about 3–6 times above normal levels. As discussed above, three modifications to the capillary bed underly barrier function: expression of tight junctions that limit paracellular leakage and loss of fenestrae and macropinocytosis, limiting transcellular pathways. Although tight junction protein structure and function are often altered with viral exposures, the maximum opening of the paracellular pathway may only be about 20 nm [147]. However, viruses have diameters of 75–100 nm. Thus, it is more likely that disruption based on the induction of vesicles with diameters of 100–150 nm participates in free virus entry into brain. Paracellular opening of the BBB could permit brain entry of viral proteins or other inflammatory molecules, which could promote vesicular modes of BBB disruption.

### 4.4. Entry of Free Virus at the Brain Barriers Is Mediated by Vesicular Pathways

As discussed above, the diameter of viruses suggests that vesicles are key to transport of viruses across the BBB. To enter non-barrier cells, viruses are known to use a variety of vesicular systems [148]. The vesicular pathways are induced by interactions between the viral attachment protein (VAP) and glycoproteins, glycolipids, or phospholipids on the cell membrane of the cell being invaded. In the case of SARS-CoV-2, the VAP is the spike protein. Although the cell membrane binding site is referred to as a receptor, the physiological function of that binding site may not be that of a receptor [149]. For example, in the case of SARS-CoV-2, the ACE2 “receptor” is a membrane bound enzyme. As such, VAP binding to the receptor does not depend on the physiologic function of the receptor to cross the BBB, but induces a process resembling adsorptive transcytosis. Adsorptive endocytosis was originally described for the BBB as a mechanism for engulfing lectin glycoproteins on the brain endothelial cell luminal membrane [150]. As a corollary, viral proteins including the VAPS can sometimes themselves cross the brain barriers.

### 4.5. Inflammation Enhances Uptake of Viruses by Brain

Inflammation is induced in response to viral infections and may also exist prior to viral infections in the case of chronic illness and/or co-infection. As discussed in the previous section, inflammation induces many alterations of BBB functions through a variety of mechanisms [13]. These changes contribute to many of the CNS-mediated symptoms noted with viral infections including cognitive impairments, depression-like behaviors, malaise, and fever. Inflammation-induced BBB changes also include changes that result in increased penetration of virus into brain. In the case of free virus entry, adsorptive transcytosis is enhanced [151]. In the case of infected immune cells, diapedesis is enhanced [145]. For HIV-1, these processes are fairly well worked out. Inflammation acting at the BEC stimulates the MAPK pathways, releasing interleukin-6 and granulocyte-macrophage colony-stimulating factor from the luminal surface of BECs, stimulating transcytosis of free virus [152]. Stimulation of the BEC CD40L receptor increases adhesion molecules through JNK-dependent mechanism, increasing the transport of monocytes across the BBB [153]. An increase in BEC adhesion molecules has also been proposed to be central to the immune cell-mediated transport into brain of Zika virus and West Nile virus as well [154,155].

Viral entry may vary with the course of neuroinflammation as well. For example, Varicella-zoster virus has been reported to be present in CSF of a small group of MS patients in the acute phase of a relapse, but absent from the CSF of MS patients in remission and in control subjects [156].

### 4.6. The Viral Attachment Protein May Bind to a Site on the Receptor Not Used by the Physiological Ligand

The VAP may or may not bind partially or entirely to the site used by the physiological ligand. The VAP-receptor interaction does not relate to the physiological function of the receptor, but induces adsorptive endocytosis. As originally described, vesicles are routed to lysosomes and subsequently returned to the luminal surface [150]. However, some vesicles are routed to other membranes, including the abluminal surface. Luminal to abluminal routing constitutes passage across the BBB (transcytosis). VAP-receptor binding may not follow classic Michaelis-Menten kinetics. Indeed, a hallmark of adsorptive transcytosis is that increasing the amount of ligand can enhance, rather than saturate, passage [157]. If the VAP binding site partially overlaps with the physiologic ligand, then the ligand may be able to induce partial inhibition of viral penetration across the BBB.

### 4.7. Some Viruses Bind to More Than One Seemingly Unrelated Receptor

HIV-1 binds to CD4, galactosyl ceramide, and mannose-6 phosphate receptors. Herpes simplex virus binds to heparan sulfate, a tumor necrosis factor-α-related receptor, nerve growth factor receptors, CD155, polio related receptor 1 and 2, and nectin 1-alpha and 2-alpha [149,158]. Rabies binds to the acetylcholine receptor, neural cell adhesion molecule, and nerve growth factor receptor [158]. Being able to bind to multiple receptors allows a virus to invade multiple tissues. For example, HIV-1 uses galactosyl ceramide to invade immune cells, but brain endothelial cells, which do not possess galactosyl ceramide [159], are invaded using the mannose-6 phosphate receptor [160]. In cases when a virus binds primarily or more avidly to a single receptor, the other binding sites are referred to as alternative receptors. Binding of the VAP can be to a single receptor or may require subsequent or simultaneous binding to co-receptors for attachment or entry [149]. Although the receptors are apparently unrelated, the receptors tend to be rich in sialic acid. Receptor recognition and species barriers are often related to variations in glycosylation patterns. Additionally, some viruses use proteins which they have acquired from the host cell as VAPS, as illustrated by paramyxoviruses viruses and herpes viruses [149]. Some coronaviruses bind to ACE2 and to other binding sites, such as CD209L [161].

### 4.8. Mechanisms of SARS-CoV-2 Entry into Brain

Currently, there is no study of the mechanisms by which SARS-CoV-2 enters the CNS. There is, however, a study on the ability of the S1 VAP to cross the murine BBB [162], at least three studies of the appearance of live virus in mouse brain after induction of a productive infection [61,66,70], and analysis of human brain tissue and/or CSF that suggests brain entry or infection can happen, as discussed in an earlier section. These studies guide the application of the principles of general viral entry into brain as they likely apply to SARS-CoV-2.

SARS-CoV-2 administered nasally results in its appearance in brain [61,66]. This study clearly establishes that the virus can enter the CNS, but does not establish whether the virus is taken up by the olfactory bulb and trigeminal nerve, is absorbed into the blood stream via the nasal turbinates, or is aspirated into lung where a productive infection then seeds the brain by hematogenous spread. Another study found that the epithelium of the olfactory bulb was massively damaged with immune cell infiltration in Syrian hamsters infected with SARS-CoV-2, but that there was no infection of the olfactory bulbs themselves [163]. The study using S1 [162] found a very small amount of S1 entering the brain after nasal administration at the level of the cribriform plate and evidence for an even smaller entry into the blood stream. By contrast, passage from blood to brain was much more robust. The most parsimonious explanation for these results is that direct spread from the nares to brain is likely a more minor route than that of blood-to-brain transport.

ACE2 is thought to play a dominant role in uptake of SARS-CoV-2 by all tissues including the brain barriers and CNS tissues. However, several studies have presented various types of results indicating that S1 could use other glycoproteins as receptors or co-receptors, including basigin, cyclophilins, dipeptidyl peptidase-4 and GRP78 [164,165,166]. In the S1 study [162], evidence suggested a role for S1 uptake into brain, but was also suggestive that other binding sites might also play a role. In contrast, ACE2 played a much larger role for uptake by lung, but little or no role for uptake by other tissues, suggesting other binding sites could be more important for their uptake. At present, our interpretation is that ACE2 is important in brain uptake, but may not be the only binding site involved. As a corollary of the S1 study, ACE2 is likely much more involved in lung uptake, but other binding sites may be key in viral uptake by other tissues.

Given the experimental design used in the S1 study [162], the vascular BBB is likely a site of entry into brain. ACE2 is expressed on the epithelial cells which comprise the choroid plexus and SARS-CoV-2 can infect those cells in vitro [167]. This suggests that the virus likely enters brain at both the vascular BBB and at the choroid plexus.

The infection study of Song et al. found very little cellular infiltrate in brain [61]. Given the experimental design of S1, it is likely S1 entry did not involve transport via immune cells. The best conclusion based on these findings is that SARS-CoV-2 can enter as free virus, but that entry via infected immune cells deserves further study.

**Figure 2:**
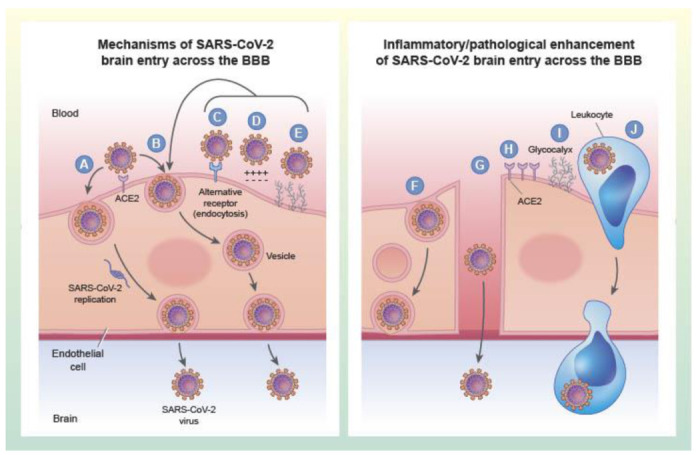
Mechanisms of SARS-CoV-2 entry into the brain across brain endothelial cells. The left panel (**A**–**E**) shows proposed mechanisms of entry via (**A**) ACE-2-dependent infection and replication inside brain endothelial cells, resulting in viral release on the brain side, (**B**) ACE2-dependent transport across the endothelial cell without replication, (**C**) receptor-mediated transport by a receptor other than ACE2, (**D**) transport mediated by adsorptive transcytosis, (**E**) transport that is mediated through interactions with the endothelial glycocalyx. The right panel (**F**–**J**) shows mechanisms by which inflammation or other pathological conditions may enhance or additionally contribute to SARS-CoV-2 brain entry. Inflammation can increase brain endothelial vesicular pathways of leakage (**F**), or formation of transendothelial channels (**G**) which may be large enough to permit viral leakage from blood. Inflammation and other diseases may contribute to upregulation of ACE2 (**H**) or other receptors that could increase viral infection and/or transport. Reduction or change in composition of the brain endothelial glycocalyx (**I**) could alter the interactions of SARS-CoV-2 with the endothelium. Finally, trafficking of immune cells across the BBB may be upregulated during inflammation or other pathological conditions (**J**), which could facilitate viral entry via a Trojan horse mechanism if the immune cell is infected.

Inflammation is a key feature of COVID-19 and is likely of a magnitude that results in both paracellular and transcellular disruption of the BBB. In the S1 study [162], activation of the innate immune system did not enhance the entry of S1 by way of adsorptive transcytosis, but did enhance S1 entry by BBB disruption, likely via the transcellular pathways. A summary of these possible routes of entry and their modifications under pathological conditions is depicted in Figure 2. There is not presently much more information on the contributions of inflammation to mechanisms of SARS-CoV-2 brain infection, but animal models of SARS-CoV-2 infection with neurotropism do exhibit both systemic and neuroinflammation [61,66,168] and therefore it is plausible that future studies could evaluate whether anti-inflammatory interventions inhibit neuroinvasion of SARS-CoV-2. Non-replicative pseudoviruses that have the structural protein components of SARS-CoV-2 could also be used as tools to more effectively study viral entry that is regulated by inflammation in the absence of confounds such as differences in systemic viral load that may also be influenced by blocking inflammation. In summary, S1 protein can clearly cross the BBB and best evidence to date suggests that SARS-CoV-2 is also likely to do so using vesicular mechanism akin to adsorptive transcytosis.

## 5. Co-Morbidities That Could Influence SARS-CoV-2 Entry into the Brain

As was discussed in previous sections, the disease that develops following SARS-CoV-2 infection can range from unnoticeable to deadly. Factors such as age, male sex, preexisting disease, and genetic risk variants have been identified that appear to increase the risk for developing severe COVID-19. We posit, based on the limited evidence in humans and animals, that brain viremia and adverse neurological outcomes are more likely to occur in those who develop severe COVID-19. In this section, we will consider how co-morbid conditions that increase risk for severe disease could also increase the ability of SARS-CoV-2 to enter or infect the brain.

### 5.1. Type I Interferons

Interferons are cytokines that interfere with viral replication and activate cells of the immune system to respond to viral infection. Interferons can be classified into three types: Type I, II, and III which engage different receptors and signaling pathways [169,170]. Recent studies have identified type I interferon signaling deficiencies in individuals with severe COVID-19 [171,172,173]. These deficiencies include autosomal loss-of-function mutations in genes upstream of IFN-α production, resulting in low levels of IFN-α in blood following SARS-CoV-2 infection [172], or production of anti-IFN-α autoantibodies [173]. However, recent work in a mouse model indicates that an inhibition of the type I interferon response prevents an inflammatory response in the lungs, but has little effect on viral replication [168]. Less is known about how interferons regulate SARS-CoV-2 brain infection or entry, although interferons have been shown to regulate brain infections of other viruses. For example, nasal inoculation of RNA (VSV) or DNA (CMV) viruses induces a widespread upregulation of type I interferon signaling, which prevents viral spread throughout the brain [174]. Interferons also regulate the permissiveness of microglia and astrocytes to measles virus infection [174]. In brain organoids infected with SARS-CoV-2, however, type I IFN signaling was not observed [61], which suggests that SARS-CoV-2 may evade host mechanisms that suppress viral spread in the brain, although this has not yet been confirmed in vivo. Type I interferons were shown to have a stabilizing effect on the BBB in context of autoimmune CNS diseases and viral infections [175,176]. Therefore, deficiencies in type I IFN responses to SARS-CoV-2 infection could, in theory, contribute to a loss of protection at the level of the BBB.

### 5.2. Apolipoprotein E

Apolipoprotein E (ApoE) plays a critical role in cholesterol metabolism and transport. In humans, there are three major alleles including APOE-ε2 (ApoE2), APOE-ε3 (ApoE3), and APOE-ε4 (ApoE4), encoded on chromosome 19. ApoE4 is associated with cardiovascular disease, atherosclerosis, and Alzheimer’s disease. COVID-19 severity is also affected by these pre-existing morbidities including dementia, cardiovascular disease, and type 2 diabetes [177]. Therefore, while ApoE can have independent effects on COVID-19 severity, it is also important to consider the implications of ApoE4 co-morbidities on SARS-CoV-2 infection.

ApoE isoform has recently been suggested to increase the risk for severity of COVID-19 infection and this can occur independently of pre-existing morbidities including dementia, cardiovascular disease, and diabetes [178]. It is thought the ApoE isoform can alter the severity of viral infection. While there have been hypotheses generated about how this might occur, the direct impact of ApoE isoform and SARS-CoV-2 has not been investigated. Many of these hypotheses have been generated based on previous studies investigating the impact of ApoE isoform on other viral infections, such as with hepatitis C or HIV-1.

ApoE is associated with the susceptibility to general viral infections [179]. ApoE can mediate viral entry by either interacting with the virus, interacting with proteins and receptors present on the host cell surface, or impacting the immune response once infection has occurred [177]. For example, ApoE can mediate hepatitis C viral binding to human hepatocyte-like cells by engaging interaction with heparan sulfate proteoglycan receptors [180]. The impact of APOE alleles on hepatitis C infection are likely viral strain- and cell-specific [181]. In another example, ApoE4 increases the susceptibility, severity, and CNS invasiveness of herpes simplex virus-1 (HSV-1) in mice and humans compared to ApoE3 [182,183,184]. Lastly, ApoE4 increases HIV-1 cell entry and expression of two copies of the ApoE4 allele results in an even more rapid HIV disease progression [185]. In addition, ApoE4 is associated with increased BBB disruption in humans [186] which could also contribute to an increased SARS-CoV-2 entry into the brain depending on the mechanism of leakage.

ApoE lipoproteins can modulate parts of the immune response by acting on T-lymphocyte activation and proliferation [187]. Based on these immunomodulatory properties, ApoE can impact the pathology of infectious diseases [188]. There is also a relationship between ApoE and cytokine production and vice versa. Cytokine levels and secretion are dependent on the APOE allele expressed [189]. For example, ApoE4 macrophages secrete higher levels of TNF-α compared to ApoE3 [190]. In addition, in response to an inflammatory stimulus, ApoE3 expressing cells produce less pro-inflammatory cytokines compared to ApoE4 [190]. Alternatively, pro-inflammatory cytokine expression can decrease the production of ApoE [191], which could have indirect effects on the beneficial action of ApoE.

Specifically related to SARS-CoV-2, computational analysis suggests angiotensin-converting enzyme 2 (ACE2), the receptor suggested to be primarily involved in mediating SARS-CoV-2 uptake, can interact with ApoE [192]. In addition, the C terminus end of the S1 protein has been shown to directly bind neuropilin-1 (NRP1) and NRP1 receptor [46]. Whether the different APOE alleles affect these interactions are currently unknown.

We have recently investigated the impact of ApoE genotype and sex on S1 BBB transport in mice [162]. *APOE* genotype affects S1 transport into liver, spleen and kidney with ApoE4 resulting in a slower transport rate. Transport of S1 is greatest in the olfactory bulb in male ApoE3 mice. There was no difference in the rate of transport into whole brain. These results suggest that enhanced uptake of S1 by some tissues could contribute to the increased risk of COVID-19. While there is previous evidence for ApoE effects on viral infections, further investigations to understand the biological mechanisms linking *APOE* genotypes and COVID-19 severity are needed.

### 5.3. Disease States That May Increase Risk of SARS-CoV-2 Entry into Brain

COVID-19 severity and mortality is increased in patients with diabetes mellitus, cardiovascular disease, and obesity. Links between these diseases with COVID-19 include effects on glucose homeostasis, inflammation, altered immune response, and activation of the renin-angiotensin-aldosterone system through the ACE2 receptor. This system contributes to blood flow, endothelial function, inflammation, insulin resistance, and vasodilation [162,193]. Therefore, disruption of this system contributes to the detrimental effects in individuals with diabetes or obesity.

At least in human monocytes, high glucose concentrations increase replication of the virus [194]. Whether the same occurs in other cells exposed to high glucose such as brain endothelial cells is unknown. Poor glycemic control predicts COVID-19 severity and increases mortality [195]. In addition, SARS-CoV-2 infection can induce loss of glycemic control. Whether the same thing happens in pre-diabetic obese individuals remains to be determined.

Hyperglycemia and obesity can affect immune function and it is known viral infection leads to increased production of pro-inflammatory cytokines, which can lead to a cytokine storm in some instances. Therefore, in patients with diabetes and obesity, the inflammatory response is further aggravated by SARS-CoV-2 infection. This inflammatory response can also affect insulin resistance, establishing a vicious cycle [196]

Of patients that underwent MRI and CT scans during COVID-19 infection, one in five showed evidence of strokes, brain bleeds, and blocked blood vessels. Half of these patients had pre-existing histories of hypertension and/or type 2 diabetes [197,198]. Currently, a challenge in interpreting the results of neuropathological associations with SARS-CoV-2 infection is that comorbid conditions such as hypertension, diabetes, and obesity may contribute to neuropathologic changes such as neurovascular damage, white matter injury, and neuroinflammation independently of SARS-CoV-2 infection [199,200,201,202]. Future work in larger patient cohorts is needed to determine whether patients with diabetes, hypertension, or other conditions increases the risk for COVID-19 associated neurologic sequalae, or whether certain CNS pathologies that arise with SARS-CoV-2 infection are more common or more severe in those with these preexisting co-morbidities.

## 6. Conclusions

In this review, we have summarized the current evidence which supports that SARS-CoV-2 may infect cells in the brain, at least in some more severe COVID-19 cases, and mechanisms by which it may enter the brain across the BBB. Human studies have variably reported the ability to detect SARS-CoV-2 in brain tissue and CSF, indicating that further studies are needed to evaluate how common or rare SARS-CoV-2 neuroinvasion is. Mouse models of SARS-CoV-2 infection suggest that brain cells, particularly endothelial cells and neurons, can be infected and/or damaged, but the mechanism of brain uptake of infectious virus in these models has not yet been determined. Studies using portions of the spike VAP of SARS-CoV-2 have shown in wild-type mice that S1 can cross the intact BBB through a mechanism of adsorptive endocytosis, suggesting that human ACE2 is not required. The olfactory route is a minimal contributor to S1 protein uptake into the brain. Future work in animal models could improve our understanding of how SARS-CoV-2 enters the brain, but data generated using transgenic ACE2 mice driven by artificial promoter systems should be interpreted carefully when considering translatability of the results to humans. SARS-CoV-2 infection may influence the functions of BECs and other components of the NVU through direct physical interactions with the virus and its proteins, or through inducing host factors such as cytokines. It is plausible that both mechanisms could influence long-term neurological outcomes in COVID-19 survivors. Co-morbidities that increase risk for severe COVID-19 may also alter aspects of BBB functions that regulate brain entry of SARS-CoV-2. In conclusion, although there is much left to be learned about the involvement of the BBB in SARS-CoV-2 infection and how the BBB may contribute to the neurologic sequalae of COVID-19, the BBB is likely involved by several mechanisms in the expression of those neurological sequalae.

## Figures and Tables

**Table 1:** Summary of studies investigating SARS-CoV-2 neurotropism and associated pathologies in humans.

Tissue Examined	Subject/Cohort Description	Post-Mortem Interval	Method of SARS-CoV-2 Detection in CNS	Detection Information	Associated Pathologies	Ref
CSF	Multiple, systematic review	Not reported	qPCR	Detected in 4/18 subjects	Not reported	[51]
Brain Biopsies				Detected in 8/34 subjects		
Brain tissue	Case report, 74-year-old Hispanic male with Parkinson’s disease; PCR positive NP swab. Febrile, hypotensive, thrombocytopenic, declining SpO2, elevated CRP, D-dimer, ferritin	Not reported	Transmission electron microscopic detection of viral-like particles	Detection of viral-like particles in frontal lobe, inside endothelial cell vesicles, and blebbing from endothelial membrane. Additionally, in neurons.	Vacuolization of neuronal cytoplasm	[55]
Brain tissue			qPCR	Detected		
CSF (post-mortem)			qPCR	Not detected		
CSF	13 subjects with severe SARS-CoV-2, NP swab confirmed and presenting with pneumonia, seizures and/or encephalopathy	N/A, subjects were alive	qPCR (LOD 181 copies/mL)	SARS-CoV-2 not detected in CSF, but verified in NP swabs, some taken on same day.	No pleocytosis in CSF except in once case of hemorrhage. 9/11 examined had abnormal MRI/CT, evidence of subcortical hypoxic/ischemic injury	[58]
CSF, brain CT/MRI	58 patients with NP swab confirmed SARS-CoV-2 and neurological manifestations, 47 had acute respiratory failure	N/A, subjects were alive	qPCR (LOD 500 copies/mL)	SARS-CoV-2 detected in 4/58 subjects; 3 below LOD	In CSF: 10 had increased WBCs, 19 had elevated albumin quotient, 21 had elevated IgG, 5–7 had elevated IL-6 and TNF-a. 36/53 subjects evaluated had CT/MRI abnormalities.	[59]
Post-mortem FFPE and frozen brain tissues	43 patients confirmed with NP swab, age range 51–94. 40 had chronic medical conditions, 13 had pre-existing neurological disease, 12 died in ICU; deaths were primarily from viral pneumonia	0–9 days (3.3 mean)	qPCR, S and N histochemistry	9/23 total had RNA detected; 9 in frozen frontal lobe, 4 in FFPE medulla oblongata. 16/40 had S and/or N detected in medulla oblongata and along cranial nerves; 14/16 S+, 7/16 N+. 21/40 had RNA or protein detected; Of 16 brains with RNA and protein measured, 8 had both, 4 had protein only, 4 had RNA only.	Brain edema (53%), Arteriosclerosis (100%), Gross macroscopic abnormalities (30%), Fresh ischemic lesions of cerebral arteries (14%), no cerebral bleeding/small vessel thrombosis, astrogliosis in olfactory bulb, basal ganglia, brainstem, cerebellum, microgliosis in brainstem and cerebellum, HL-DR in subpial and subependymal regions.	[60]
Post-mortem FFPE brain sections	Three subjects who died of severe COVID-19; respiratory failure, on ventilator, PCR positive postmortem lung. All had comorbidities (hypertension, obesity, or kidney transplantation)	Not reported	S1 histochemistry	S1 detected in cortical neurons and endothelial cells; positive viral staining detected around infarcts in one patient	No leukocyte infiltration in regions with S1 staining	[61]
Tissue Examined	Subject/Cohort Description	Post-Mortem Interval	Method of SARS-CoV-2 Detection in CNS	Detection Information	Associated Pathologies	Ref
FFPE brain tissue sections	18 subjects with PCR-confirmed COVID-19 age 48–90. Neurologic sequalae: myalgia (3), headache (3), loss of taste (1). Co-morbidities: diabetes (12), hypertension (11), cardiovascular disease (5), hyperlipidemia (5), chronic kidney disease (4), prior stroke (4), dementia (4), anaplastic astrocytoma (1)	20–102 h	qPCR for SARS-CoV-2 nucleocapsid mRNA and histochemistry for N protein: frontal lobe/olfactory nerve and medulla for all patients; cingulate/corpus collosum, hippocampus, occipital lobe, basal ganglia, thalamus, cerebellum, midbrain, pons were additionally tested in two subjects	Equivocal detection (<5 copies/cm^3^) in 5/10 and 4/10 sections from the two subjects with 10 regions assessed; in 16 subjects with 2 regions assessed, 5 subjects had > 5 copies/cm^3^, 8 subjects had equivocal detection, and 3 subjects had no detectable mRNA. N protein not detected.	All subjects had evidence of acute hypoxic changes in the cerebrum and cerebellum, no microscopic abnormalities of olfactory bulb/olfactory tracts, neuronal loss in hippocampus, cerebrum and cerebellum but no thrombi or vasculitis. Perivascular lymphocyte foci detected in 2/18 subjects.	[62]
Post-mortem FFPE and frozen brain tissue	19 patients confirmed with NP swab, age range 5–73	5–368 h	qPCR, RNAscope	SARS-CoV-2 not detected	Out of 19: Vascular pathology (11), perivascular infiltrates (13), acute hypoxic/ischemic neuronal damage (6), no path findings (2)	[63]
CSF, brain CT/MRI	Case report, 55-year-old previously healthy woman with PCR-confirmed COVID-19, pulmonary ground glass opacities. Found unresponsive in bed without hemodynamic or respiratory issues.	N/A, patient survived and was discharged.	qPCR	CSF was collected on day 9, 12, 14, and 26 from first symptoms, SARS-CoV-2 detected only on day 14 (cycle threshold = 34.29)	CSF day 9: no pleocytosis, but elevated albumin. CSF day 12: no pleocytosis, albumin normal, IgG elevated without autoantibodies. Elevated GFAP, NFL, tau, and IL-6. CSF day 14: further increases in NFL and tau and reductions in GFAP and IL-6, increases in CSF total protein, and appearance of oligoclonal bands. CT and MRI: symmetrical hypodensities in thalami that progressed to midbrain; acute necrotizing encephalitis	[64]

**Table 2:** Summary of studies investigating SARS-CoV-2 neurotropism and associated pathologies in mice.

Model	Tissue Examined	Method of SARS-CoV-2 Detection	Detection Information	Associated Pathologies	Ref
K18-hACE2 1.5 × 10^6^ PFU intranasal	Whole brain homogenate	qPCR	Yes- day 2, 4 and 7 post-infection all mice		[61]
	Whole brain homogenate	Viral titers	Yes- day 2, 4 and 7 post-infection all mice		
	iDISCO cleared brains	Immunolabeling of N protein/ light sheet microscopy	Yes- Forebrain neural cells, sensory cortex, dentate gyrus, globus pallidus, cortical layer IV, not cerebellum, not endothelium day 7 post-infection	Remodeling of vasculature found in proximity to virus	
K18-hACE2 2.5 × 10^4^ PFU intranasal	Whole brain homogenate	qPCR	Yes- day 2, 4, and 7 post-infection all mice		[66]
	Whole brain homogenate	Viral titers	Yes- day 7 for 4/10 mice tested, but not on day 2 or 4.		
	FFPE Brain sections	qPCR (non-fixed side)	Sections from brains with high or low/no viral load compared for pathologic changes	No/low SARS-CoV-2 brains had minimal/no brain pathology, SARS-CoV-2 infected brains had meningeal inflammation, leukocyte extravasation to parenchyma and microglia activation	
hACE2 humanized mouse 4 × 10^5^ PFU intranasal	Whole brain homogenate	qPCR	Yes- day 6 post-infection all mice, absent in control mice		[70]
